# Clinical features of X linked juvenile retinoschisis in Chinese families associated with novel mutations in the *RS1* gene

**Published:** 2007-06-07

**Authors:** Xiaoxin Li, Xiang Ma, Yong Tao

**Affiliations:** Department of Ophthalmology, People's Hospital, Peking University, Beijing, P R China

## Abstract

**Purpose:**

To describe the clinical phenotype of X linked juvenile retinoschisis (XLRS) in 12 Chinese families with 11 different mutations in the XLRS1 (*RS1*) gene.

**Methods:**

Complete ophthalmic examinations were carried out in 29 affected males (12 probands), 38 heterozygous females carriers, and 100 controls. The coding regions of the *RS1* gene that encodes retinoschisin were amplified by polymerase chain reaction and directly sequenced.

**Results:**

Of the 29 male participants, 28 (96.6%) displayed typical foveal schisis. Eleven different RS1 mutations were identified in 12 families; four of these mutations, two frameshift mutations (26 del T of exon 1 and 488 del G of exon 5), and two missense mutations (Asp145His and Arg156Gly) of exon 5, had not been previously described. One non-disease-related polymorphism (NSP): 576C to T (Pro192Pro) change was also newly reported herein. We compared genotypes and observed more severe clinical features in families with the following mutations: frameshift mutation (26 del T) of exon 1, the splice donor site mutation (IVS1+2T to C),or Arg102Gln, Arg209His, and Arg213Gln mutations.

**Conclusions:**

Severe XLRS phenotypes are associated with the frameshift mutation 26 del T, splice donor site mutation (IVS1+2T to C), and Arg102Gln, Asp145His, Arg209His, and Arg213Gln mutations. The wide variability in the phenotype in Chinese patients with XLRS and different mutations in the *RS1* gene is described. Identification of mutations in the *RS1* gene and expanded information on clinical manifestations will facilitate early diagnosis, appropriate early therapy, and genetic counseling regarding the prognosis of XLRS.

## Introduction

X-linked retinoschisis (XLRS) is one of the most common causes of juvenile macular degeneration in males [1], with a worldwide prevalence of 1 in 15,000-25,000 men [2]. This X-linked trait affects only men; female carriers rarely have vision morbidity [3,4]. XLRS is characterized by microcystic-like changes of the macular region of the retina and schisis, or splitting within the inner retinal layers, leading to visual deterioration [5,6]. Peripheral retinal lesions are also present in about 50% of cases [6]. The clinical course generally causes a moderate decrease in visual acuity [5], but more advanced stages are complicated by vitreous hemorrhage, retinal detachment (RD), and neovascular glaucoma [7], which may induce severe loss of vision. Severely affected male infants may be blind at birth from bilateral RD [5]. Approximately 50% of XLRS patients also have a decrease in visual field. The brief-flash electroretinograms (ERGs) of most affected males exhibit normal or near normal a-waves characteristic of photoreceptor function but often substantially reduced b-waves, originating from inner retinal cell activity [8].

The gene responsible for XLRS was identified by positional cloning [9]. The XLRS1 or *RS1* gene contains six exons that encode a small, 224-amino-acid protein, with an N-terminal secretory leader peptide sequence [10,11] and a discoidin domain in exons four to six that is highly conserved across species [12]. Discoidin domains are found in a large family of secreted or membrane-bound proteins and have been implicated in cell adhesion and cell-cell interactions [13]. Numerous disease-causing mutations in *RS1* gene have now been recognized [14] (DMD).

The majority of these are missense mutations, although nonsense mutations, deletions, insertions, and splice site mutations have all been found. The correlation between the phenotype and genotype of XLRS remained unclear according to the reports [15,16]. The clinical features in families from several countries with defined mutations in the *RS1* gene have been reported [3,17-20].

In this study, we describe Chinese patients who have mutations in their *RS1* gene, and we examine their genotype-phenotype correlations. Four of these mutations have not been previously reported.

## Methods

### Clinical studies

The research protocol was approved by the ethics review board of the Peking University School of Medicine. The study procedures were carried out in accordance with institutional guidelines and the Declaration of Helsinki.

**Figure 1:**
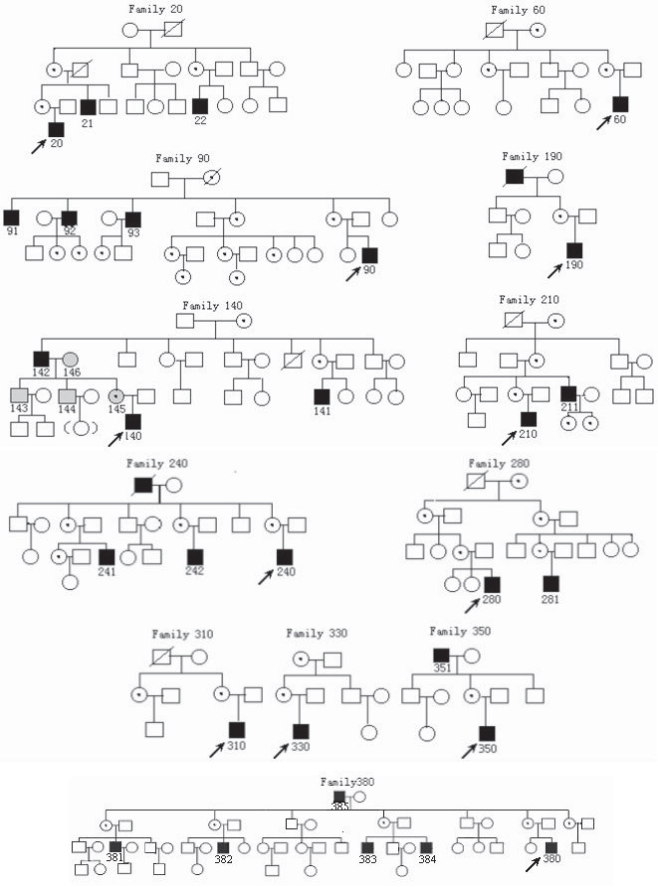
Pedigrees of families with X linked juvenile retinoschisis and identified mutations in the *RS1* gene. Dark-shaded boxes represent family members with X-linked juvenile retinoschisis, while light-shaded boxes and circles mark those with nondisease-related polymorphisms. Circles with a dot denote obligate carriers. Arrows point to probands. Slashed circles or boxes are deceased family members.

Twelve families with XLRS (Figure 1) were recruited for this study. Ophthalmic examinations were performed in 29 affected males, 38 heterozygous females carriers, and 100 controls (with normal vision and without any eye diseases male, not the members of XLRS families). The mean age was 27 years (SD 19) for the males, 45 years (SD 11) for the females, and 33 years (SD 16) for the controls. The examinations included best-corrected visual acuity (BCVA), slit lamp biomicrocopy, fundus examinations and photography, fluorescein angiography, A and B scan, standardized echography, optical coherence tomography (OCT) and single-flash electroretinograms (ERG). ERG results from the XLRS patients were compared with those of 48 male controls (48 controls for ERG examination) subjects with normal vision, whose ages ranged from 11-43 years (mean age 32 years with SD 15). The "reduced" ERG b-wave defined as values below mean-2 SD of controls values. The diagnostic criteria in XLRS1 male patients included macular abnormalities defined as typical foveal schisis, blunted foveal reflex, pigmentary demarcation lines or retinal pigment atrophy with or without peripheral retinoschisis (RS), reduced ERG b-wave, and a history of bilateral visual impairment since childhood [1,5]. To examine the relationship between axial length and refractive error in patients with X-linked retinoschisis. The axial length and refractive error were measured in 51 eyes of 29 patients. The patients were divided into two groups: a juvenile group with ages <13 years (9 eyes) and an adult group with ages greater than or equal to 13 years (42 eyes). The axial length of the 40 eyes of 40 adult men without eye diseases whose refractive error ranged from ±1.0 diopter served as controls.

### Molecular genetic studies

Informed consent allowing blood and eye examination was obtained from all subjects. Genomic DNA was isolated from peripheral white blood cells using a Blood DNA Isolation Kit, PureGene (Gentra Systems, Minneapolis, MN), which was used as the template to amplify the *RS1* gene. All primers were procured from Sigma Genosis (Dalian, China). All exons (exon one to six) of the *RS1* gene were amplified by polymerase chain reaction (PCR) using previously reported primers [9]. PCR products were purified with QIAquick PCR Purification Kit (Qiagen K.K., Dalian, China) and used as the template for sequencing. Purified PCR products were sequenced on an automated sequencer (ABI Prism 3100 Genetic Analyzer, Applied Biosystems; Takara Co., Dalian, China).

## Results

Eleven different *RS1* mutations were identified in the 12 participating XLRS families (Table 1). Eight of these mutations were missense mutations, and nine were clustered in exons four, five, and six encoding the discoidin domain. One frameshift mutation (26 del T) in exon 1 (Patients 90, 91, 92, 93), and one rare splice donor site mutation (IVS1+2T to C) of the exon 1 and intron 1 junction (Patient 330) were also identified: patients with these two mutations had severe clinical features. To the best of our knowledge, four of these mutations have not been previously described, namely, Asp145His, Arg156Gly and frameshifting deletion (26delT, 488 del G) mutations of exon 5.

**Table 1:** Identification of X linked juvenile retinoschisis 1 mutations in the X-linked juvenile retinoschisis pedigrees.

**Pedigree**	**Exon**	**Mutation**	**Predicted amino acid change**	**Predicted effect**
90	1	26delT	frameshifting deletion	frameshift*
330	IVS1	52+2>T-C	Del	splice donor site
280	4	217T>C	Ser73Pro	missense
310	4	305G>A	Arg102Gln	missense
60	5	433G>C	Asp145His *	missense
380	5	466G>A	Arg156Gly *	missense
190	5	488delG	frameshifting deletion	frameshift*
140	6	576C>T	Pro192Pro*	NSP
140	6	598C>T	Arg200Cys	missense
20	6	626G>A	Arg209His	missense
240	6	638G>A	Arg213Gln	missense
210	6	638G>A	Arg213Gln	missense
350	6	667T>C	Cys223Arg	missense

Summarized are the DNA outcome of *RS1* mutations in Chinese X linked juvenile retinoschisis pedigrees. The following abbreviations were used: Ser is serine (Ser), Pro is proline (Pro), Arg is arginine (Arg), Gln is glutamine (Gln), Asp is aspartic acid (Asp), His is histidine (His), Gly is glycine (Gly), Trp is tryptophane (Trp), Cys is cysteine (Cys), and NSP is non-disease-related polymorphisms (NSP). The asterisk (*) indicates new mutation.

The clinical data of patients are reported in Table 2. In the 29 XLRS male patients, the mean age at disease onset was 5.5 (SD 1.4) years, and the initial symptoms were photophobia, squint, nystagmus, and reduced visual acuity (VA). In a few cases, the patients were asymptomatic and diagnosed after ophthalmological examination. BCVA varied from 20/20 to light perception; the refractive spherical errors (see Table 3) ranged from -7.25 to +7 diopters with mean values of +0.89 (SD 2.71). Hypermetropia in our patients, as with other studies [6,21,22], was the most frequent refractive error; the mean value of the axial length calculated with immersion standardized A-scan echography was 21.9 (SD 1.7).

**Table 2:** Clinical data of X linked juvenile retinoschisis families.

**Pedigree**		**Mutation**	**Age (years**	**Age at onset (years)**	**R/L**	**VA**	**Spherical error (D)**	**Axial length (mm)**	**Vitreous abnormalities Ophthalmoscopy**	**Macular abnormalities**	**Peripheral RS**	**Retinal detachment**
**Number**	**Subject**											
90	90	L9CfsX20*	17	4	R	enucleation						NVG before
												enucleation
					L	20/200	3.25	21.6	vitrectomy	foveal schisis	yes	PVR+ VH
	91	L9CfsX20*	62	5	R	Hm	5	21.3	vitreoretinal traction	retinal pigment	yes	"severe PVR,"
									falciform fold,	macular atrophy,		VH+ strabismus
										bone spicule		
										pigmentation		
												
					L	FC	1	20.7	vitreoretinal traction	retinal pigment	yes	"severe PVR,"
										atrophy,		VH
	92	L9CfsX20*	55	6	R	20/100	1.5	--	vitreoretinal traction	foveal schisis	yes	yes
					L	20/100	0.75	--	vitreoretinal traction	foveal schisis	yes	yes
	93	L9CfsX20*	50	5	R	20/40	0.5	23.4	vitreoretinal traction	foveal schisis	yes	no
					L	20/100	0.75	22.1	Vitreoretinal traction	foveal schisis	yes	yes
									falciform fold,	macular atrophy,		
										pigmentation		
												
330	330	52+2 T->C	19	6	R	20/200	-7.25	24.1	vitreoretinal traction	foveal schisis,	yes	RD+PVR+ VH
									falciform fold,	bone spicule		
										pigmentation		
					L	20/200	-7	24.5	vitreoretinal traction	retinal pigment	yes	yes
									falciform fold	atrophy,		
									vitreoretinal traction	pigmentation		
												
280	280	Ser73Pro	20	6	R	20/40	1	22.6	falciform fold,	foveal schisis	no	no
					L	20/40	1	22.2	falciform fold,	vitreoretinal	no	no
										foveal schisis		
									vitreous veils	traction		
	281	Ser73Pro	21	6	R	20/60	2	22	vitreous veils		no	no
					L	20/40	1.75	22.3	vitreous veils	foveal schisis	no	no
												
310	310	Arg102Gln	15	4	R	NLP	0.5	22.6	vitrectomy	--	yes	RD+ surgery
												(1994) eye
												atrophy+ strabismus
					L	20/100	-1.5	23	Vitreoretinal traction	foveal schisis	yes	RD+PVR+ VH
										retinal pigment		#NAME?
										atrophy		
												
60	60	Asp145His*	16	4	R	20/100	3	21.6	vitreous veils,	foveal schisis	yes	Yes
									vitreoretinal traction	pigmentation		
					L	20/50	1.5	22.7	vitreous veils,	foveal schisis	yes	no
									vitreoretinal traction			
190	190	Trp163X*	9	4	R	20/50	6	20.4	vitreous veils	foveal schisis	no	no
					L	20/50	7	20.5	vitreous veils	foveal schisis	no	no
												
140	140	Arg200Cys	5	4	R	20/30	0.5	21.5	vitreous veils	foveal schisis	no	no
					L	20/30	0.5	21.3	vitreous veils	foveal schisis	no	no
	141	Arg200Cys	32	--	R	20/20	--	--	--	--	no	no
					L	20/20	--	--	--	--	no	no
	142	Arg200Cys	67	--	R	20/20	--	23	no	no	no	no
					L	20/20	--	23	no	no	no	no
20	20	Arg209His	4	2	R	20/100	3	20.5	vitreous veils	foveal schisis	yes	RD+ PVR+ VH
					L	enucleation	--	--	--	--	--	NVG after
												virectomy
	21	Arg209His	21	5	R	20/50	-2	23	vitreous veils	foveal schisis	no	no
					L	20/100	-4	23.5	Vitreoretinal traction	foveal schisis	no	no
	22	Arg209His	16	--	R	20/500	--	23.9	Vitreoretinal traction	foveal schisis	yes	yes
					L	20/200	--	22.5	vitreous veils	foveal schisis	yes	yes
									retinal traction			
240	240	Arg213Gln	18	4	R	20/60	4	20.9	Vitreoretinal traction	foveal schisis	yes	yes+ nystagmus
										Bone spicule		
										pigmentation		
					L	FC	4.5	20.6	Vitreoretinal traction	foveal schisis,	yes	RD+ VH+
												nystagmus
										retinal pigment		
										atrophy		
	241	Arg213Gln	9	5	R	20/90	2	20.9	Vitreoretinal traction	foveal schisis	yes	yes
										Bone spicule		
										pigmentation		
					L	20/60	1	20	vitreoretinal traction	foveal schisis,	yes	yes
									falciform fold,	Bone spicule		
										pigmentation		
	242	Arg213Gln	19	5	R	20/90	3	21	vitreous veils,	foveal schisis	yes	yes
									vitreoretinal traction	macular atrophy		
									falciform fold,			
					L	20/100	4	20.8	vitreous veils,	foveal schisis	yes	yes
									retinaltraction	macular atrophy		
210	210	Arg213Gln	15	4	R	20/100	0.5	21.3	vitreoretinal traction	foveal schisis	yes	yes
					L	20/100	1	21	vitreoretinal traction	foveal schisis	yes	yes
	211	Arg213Gln	27	--	R	20/60	1.5	21.5	vitreoretinal traction	foveal schisis	yes	no
					L	20/60	1	21.2	vitreoretinal traction	foveal schisis	yes	no
350	350	Cys223Arg	29	6	R	20/40	-2.5	23.2	vitreous veils	foveal schisis	no	no
					L	20/60	-4	23.5	vitreous veils	foveal schisis	no	no
	351	Cys223Arg	79	--	R	20/80	1.5	22.2	vitreous veils	foveal schisis	no	no
					L	20/80	1	22.5	vitreous veils	foveal schisis		
												
380	380	Arg156Gly	22	7	R	20/100	0.5	20.4	vitreoretinal traction	foveal schisis	no	no,+ strabismus
					L	20/50	1.5	20.2	vitreous veils	foveal schisis	no	no,+ strabismus
	381	Arg156Gly	35	6	R	20/50	1.5	-	vitreous veils	foveal schisis	no	no
										pigmentation		
					L	20/50	1	-	vitreous veils	foveal schisis	no	no
										pigmentation		
	382	Arg156Gly	29	5	R	20/40	-1	-	vitreous veils	foveal schisis	no	no
					L	20/50	0.5	-	vitreous veils	foveal schisis	no	no
	383	Arg156Gly	30	7	R	20/40	1	-	Vitreoretina traction	foveal schisis	no	no
					L	20/30	0.5	-	Vitreoretinal traction	foveal schisis	no	no
	384	Arg156Gly	19	6	R	20/40	0.5	-	vitreous veils	foveal schisis	no	no
					L	20/30	-0.5	-	vitreous veils	foveal schisis	no	no

Summarized are the clinical data corresponding to different *RS1* mutations in Chinese X linked juvenile retinoschisis pedigrees. RS indicates retinoschisis and PVR refers to proliferative vitreoretinopathy. VH represents vitreous hemorrhage; RD represents retinal detachment; NVG represents neovescular glaucoma; PVR represents proliferative vitreoretinopathy. Blank sections means no data available.

**Table 3:** The means and standard deviations of the refractive error and axial length in each group refractive error axial length.

**Subjects group**	**Refractive error (D)**	**Axial length (mm)**
All patients (51 eyes)	0.89±2.71	21.93±1.67
Juvenile patient group (9 eyes)	2.86±2.67	21.28±1.23
Adult patient group (42 eyes)	0.55±2.61*	22.11±1.10*
Normal adult group (40 eyes)	-0.26±0.64*	23.83±0.53*

Table summarizing refractive data and axial length of Chinese X linked juvenile retinoschisis families. Asterisk is p<0.01 Mann-Whitney U-test. Axial length in each group refractive error represents D and axial length represents mm.

**Figure 2:**
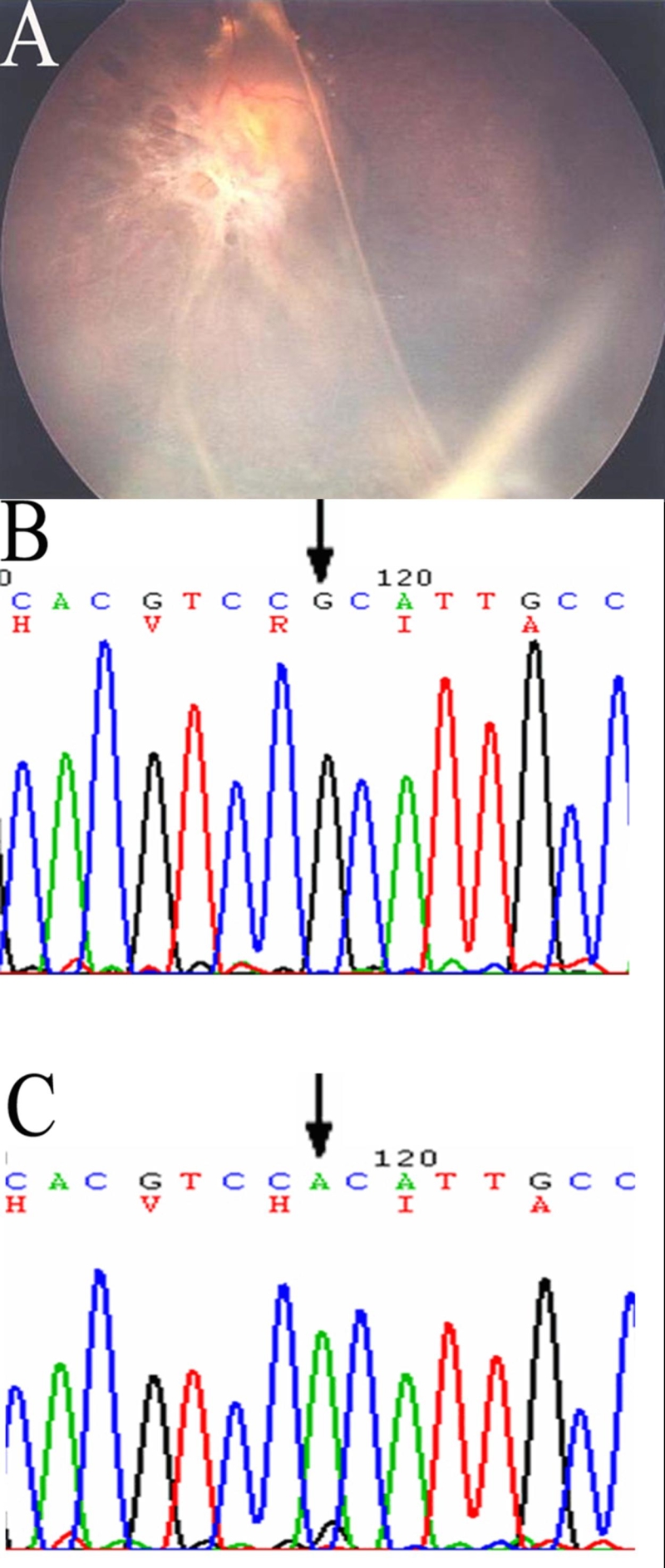
Fundus photographs and DNA sequence in Patient 20 of Family 20. Fundus of left eye (**A**) showing severe foveal and peripheral retinoschisis with tractional retinal detachment and proliferative vitreoretinopathy. DNA sequencing examination revealed Arg209His mutation -626G->A of exon 6 in normal control (**B**) and Patient 20 (**C**).

**Figure 3:**
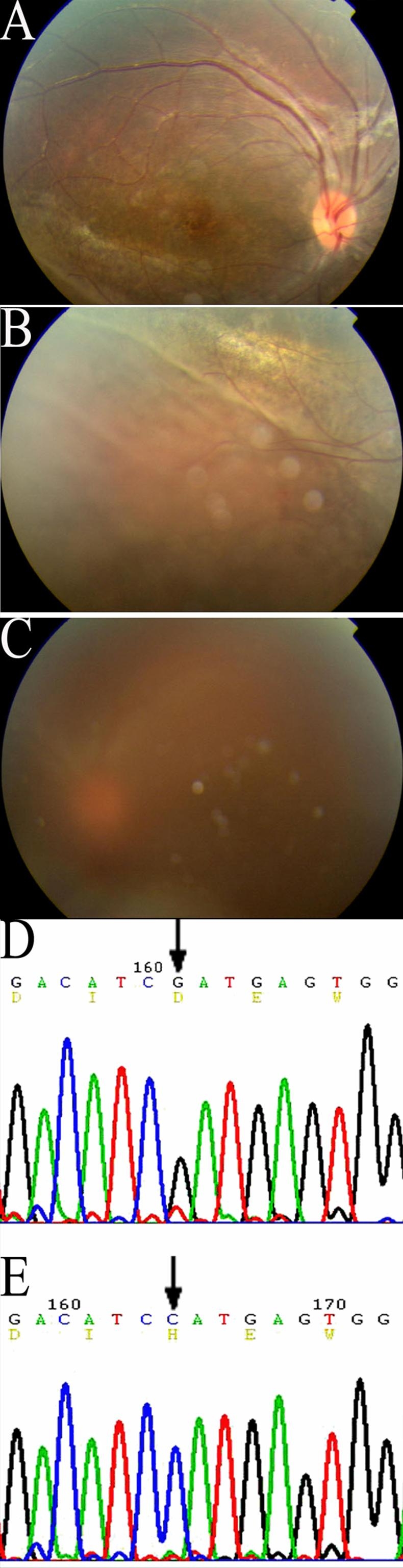
Fundus photographs and DNA sequencing in Patient 60 of Family 60. Fundus of right eye (**A**, **B**) showing cystoid-like maculopathy and peripheral retinoschisis. The left eye underwent vitrectomy with comobined sclera buckling because of retinal detachment and vitreous hemorrhage (**C**). Peripheral retinoschisis was found during the operation. DNA sequencing showed missense mutation -433G->C, Asp 145 His of exon 5 in normal control (**D**) and Patient 60 (**E**).

**Figure 4:**
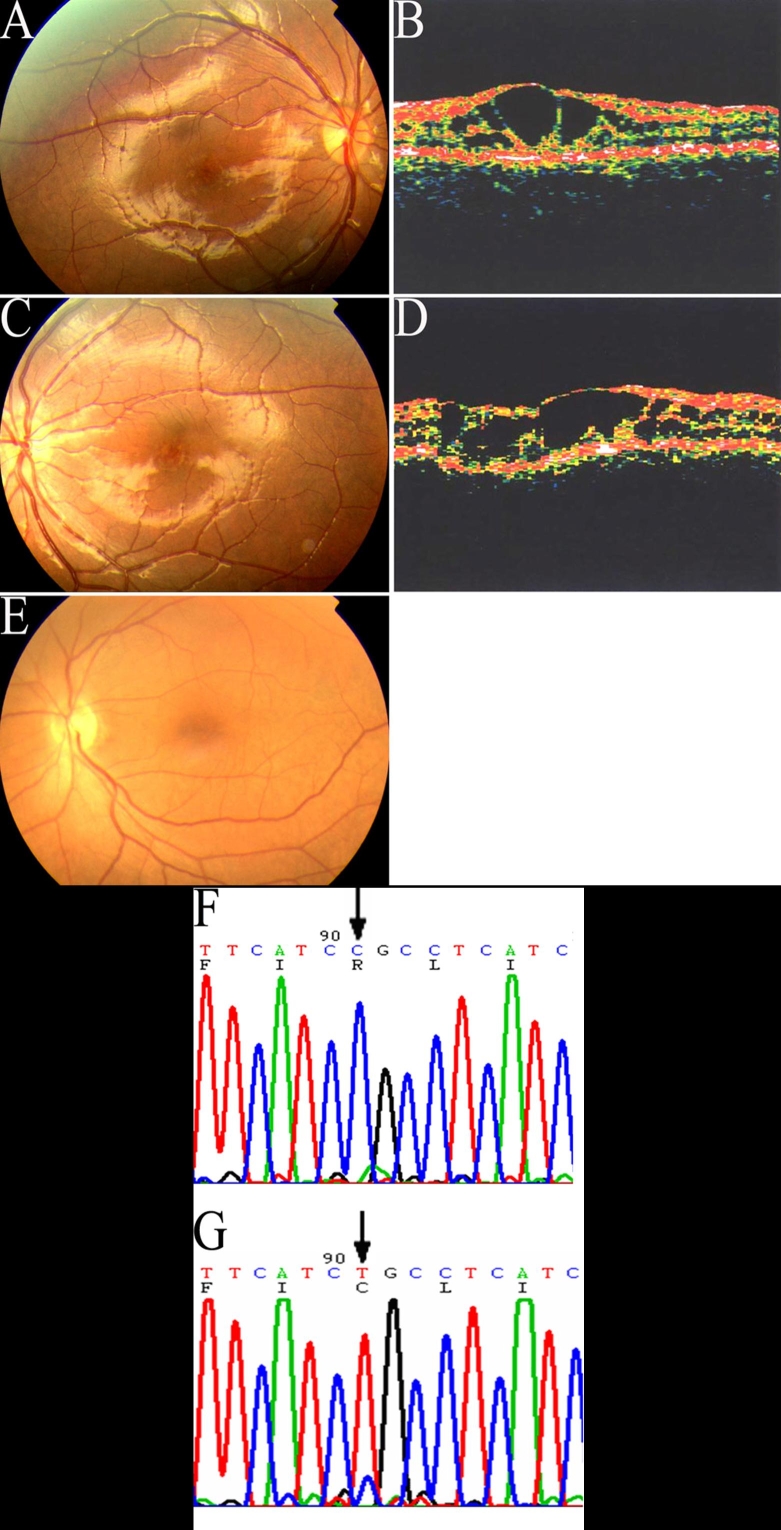
Fundus photographs, optical coherence tomography images, and DNA sequencing in Proband 140, and his grandfather, (Patient 142) of Family 140. The fundi of right (**A**) and left (**B**) eyes of Patient 140 showing cystoid-like maculopathies and golden-yellow reflex. Optical coherence tomography images of his right (**C**) and left (**D**) eyes revealed splitting had occured in the inner retina around the fovea. A normal fundus appearance (**E**) can be seen in his grandfather (Patient 142) with the same mutation. When ompared with the normal control (**F**), DNA sequencing showed the Arg200Cys mutation (598C->T) of exon 6 in Proband 140 (**G**).

Ophthalmoscopy revealed a marked interfamilial and intrafamilial variability of the fundus (Figure 2, Figure 3, and Figure 4). All but one (Patient number 142) had foveal RS. Vitreo-retinal abnormalities included vitreous veils in 25 eyes (49.0%), vitreoretinal tractions in 28 eyes (54.9%), and falciform folds in six eyes (11.5%). Macular abnormalities were present in all affected patients except one (Patient 142). All but one patient (patient 142) showed a typical foveal schisis, a cystic-like stellate alteration. In addition, ophthalmic exam revealed macular atrophy in ten eyes (19.6%), macular scarring in eight eyes, and healthy macula in three eyes. Macular abnormalities were not related to any genotype. The presence of bone spicule pigmentation was evident in 13 eyes (25.5%). Peripheral RS was evident in the temporal sector in 26 of the 51 eyes studied (51.0%). RD was present in 23 of the 26 eyes (88.5%) while vitreous hemorrhage was seen in seven (13.7%). There were 25 (49.0%) severe RS eyes with peripheral retinoschisis, RD and vitreous hemorrhage. Strabismus and neovascular glaucoma was seen in five (right eye of patient 310, both eyes of patient 380, both eyes of 240) and two eyes (one eye of patient 90 and patient 20), respectively. Two eyes from two patients were enucleated because of neovascular glaucoma secondary to RS with proliferative vetroretinopathy. These two patients came from families (pedigree 20 and 90) who had the Arg209His and frameshift (26 del T) mutations, respectively. Most of the male patients came to our department with poor visual acuity from RD. These patients also had vitreous hemorrhage, or strabismus and were seeking surgical treatment. Among the total 29 male patients, the five male patients (ten eyes) with poor VA were once misdiagnosed for amblyopia (two eyes), cataract (one eye), retinal detachment (five eyes) without RS, and other fundus diseases (two eyes).

ERG was performed on 14 patients (Table 4). In all pedigrees the typical response to white single flash was a reduction of the b-wave amplitude and a relative preservation of the a-wave amplitude, causing a reduced the ratio of amplitude of b-wave to a-wave (b/a) ratio. The b/a ratio was reduced (<1.2) in 12 patients (85.7%) while two patients (14.3%) had a reduction in both the a-wave and b-wave amplitudes, with a normal b/a ratio. Moreover, the amplitude of the a-wave was reduced in four RD patients. This reduction did not appear to be related to disease duration. The maternal grandfather (Patient 142) of proband (Patient 140), had normal b-wave and a-wave amplitude, showing a normal b/a ratio of 2.2, and also normal fundus appearance, but had the Arg200Cys mutation of exon 6.

**Table 4:** Electrophysiological data of X linked juvenile retinoschisis families.

				**Scotopic (white flash)**		
**Pedigree**	**Subject**	**Mutation**	**Age**	**b-wave (μV)**	**a-wave (μV)**	**b/a ratio**
90	90	L9CfsX125*	17	125	302	0.4
330	330	52+2 T->C	19	42.5	68.4	0.6
20	20	Arg209His	4	125	240	0.5
310	310	Arg102Gln	15	64.3	116	0.6
60	60	Asp145His*	16	191	238	0.8
190	190	Trp163X*	9	219	254	0.9
140	140	Arg200Cys	5	242	269	0.9
	142	Arg200Cys	67	281	128	2.2
240	240	Arg213Gln	18	65	149	0.4
	241	Arg213Gln	9	155	302	0.5
210	210	Arg213Gln	15	45.9	76.5	0.6
	211	Arg213Gln	27	80.8	68.6	1.2
350	350	Cys223Arg	29	208	204	1.1
380	380	Arg156Gly	22	96.7	109	0.89
				354(103)*	197(58)*	1.8(0.6)*

Table summarizing the electrophysiological data of some Chinese X linked juvenile retinoschisis patients. Asterisk is mean values (2 standard deviation [SD]) in controls.

## Discussion

Many mutations in the RS1 gene have been identified [14,23-26], but there are limited clinical data relating to the different genotypes [14,21,27]. This study examined RS1 gene mutations from Chinese families and the report regarding evaluated genotype-phenotype correlation on RS1 in Chinese patients. We found four mutations and one non-disease-related polymorphisms (NSP) not previously described [14].

In the novel frameshift mutations (26 del T) of pedigree 90 XLRS family, we observed typical foveal RS with bilateral peripheral RS in all four affected male patients of this family, and with RD in seven of the eight eyes of these four male patients. The mutation of 26 del T of exon 1, cause the frameshift mutations from amino-acid 9 (L9C), loss of a 115 amino acids long fragment at C-terminus and synthesis of an aberrant peptide from amino acid 9 to 114 followed by a premature stop, which caused the dramatic change of structure with severe type of RS. One rare splice donor site mutation (IVS1+2T to C) at the junction of exon 1 and intron 1 was also identified in this study. The patient (Patient 330) had severe foveal and peripheral RS and RD in both eyes, which required surgical intervention. A T-to-C substitution at the 5' splice donor site of intron 1 would be expected to completely block the splicing of intron 1 from the primary transcript, thus preventing formation of functional mRNA. This splice donor site mutation would also be expected to produce only severely truncated proteins without normal function, thus resulting in severe clinical features we showed herein. Our results suggest that severe cases of XLRS may be associated with upstream mutations (exons 1-3) in the RS1 gene [18], which prevent the formation of functional protein.

Families with either the Asp145His, Arg102Gln, Arg209His and Arg213Gln mutations had clinically severe RS features as compared to the genotypes of other mutations. Patient 60, who had the Asp145His mutation, showed typical foveal RS with peripheral RS in both eyes, and RD in the left eye. This patient underwent vitrectomy with combined sclera buckling. This patient with an Asp145His mutation of exon 5 showed severe RS with extensive retinal impairment. Both Patients 20 and 22 from pedigree 20 with Arg209His mutation had typical severe clinical feature with foveal RS, bilateral peripheral RS and RD in both eyes, while Patient 21 from the same family, had only mild foveal RS. The variation within this family suggested that additional factors, perhaps other genetic influences (i.e. unique environmental factors), might contribute to disease severity.

In the family with another two novel mutations (frameshifting deletion 488delG and Arg156Gly) and families with Ser73Pro, Arg200Cys, and Cys223Arg mutations we observed a mild RS clinical pictures compared with the genotypes of other mutations. In the family (pedigree 280) with Ser73Pro mutation, we evidenced the typical mild type of RS. But unlike reported by Hayashi et al. [27] that the same Ser73Pro mutation with severe clinical feature of XLRS in a Japanese patient, suggesting a variation of interfamilial phenotype with the same mutation. The phenotype associated with the Cys223Arg genotype showed a similar mild clinical picture in proband and his maternal grandfather. The RS1 discoidin domain, which extends from amino acids 63-219 [14], has been considered critical for RS1 function. This cysteine at 223 has not been observed in any of the other 28 known discoidin domain-containing proteins. Thus the Cys223Arg mutation does not appear to destroy the structure or function of RS protein [11]. It is not yet clear whether the five residues in the extreme carboxyl terminal region of the protein are an integral part of the discoidin domain or whether they contribute in some other fashion to RS1 protein interactions.

ERGs in juvenile XLRS has been shown to manifest a reduction in b-wave amplitude and a relative preservation of the a-wave on single white-flash stimulation, leading to a reduction in the b/a ratio. In this study, the b/a ratio was in the lower range, or was less than the b/a ratio in normal subjects. Patient 142 had a b/a ratio higher than 2.0. This patient had subtle macular changes as well and would have been hard to diagnose had it not been for our molecular genetics studies, which confirmed mutations in the *RS1* gene.

Flash ERG produced a negative response in all patients examined except for patient 142 who had minor macular change. Miyake and colleagues [28] reported that, despite a reduced b-wave, the a-wave was normal in early stages of XLRS, whereas it was reduced in later stages. They suggested that photoreceptors were not the primary target in the pathogenesis of this disease. However, in our study, patients 330 and 310 who were 19 years old and 15 years old, respectively, showed reduction in their a-waves and b-waves. Both these males had peripheral RS with RD. It is possible that early progression of disease in these patients is related to the particular mutations, in their RS1 gene. We further noticed that Patient 142, who was 67 years old had mild RS as evidenced by fundus appearance and normal ERG recordings. Thus, our results suggest that these variations seem to be partially independent of patient age. Moreover, the severity of the RS phenotype is partially related to the particular mutation and the position of the mutation of the RS1 gene. Surprisingly, the results of the flash ERG were relatively correlated with the severity of the clinical feature of XLRS in all our affected male patients. Thus, the ERG should be considered crucial for diagnosis XLRS disease.

The individuals in our study appeared to be more severely affected as compared with other reported cases. The reason for this is that most patients were referred to us for surgical treatment, for RD, as well as vitreous hemorrhage, or had proliferative vetroretinopathy, and many of them had been misdiagnosed earlier in their clinical course. Thus, when boys with visual impairment undergo ophthalmologic examination, it is important to complement it with a full-field ERG, since the ophthalmoscopic appearance in XLRS is variable. The combination of full-field ERG and molecular genetics makes clinical diagnosis of XLRS possible early in the course of the disease.
